# The effect of providing Medicare Advantage enrollees diagnosed with cancer additional time to reassess enrollment

**DOI:** 10.1093/haschl/qxaf131

**Published:** 2025-06-27

**Authors:** Emma M Achola-Kothari, Stacie B Dusetzina, John A Graves, David G Stevenson, Laura M Keohane

**Affiliations:** Vanderbilt University School of Medicine, Department of Health Policy, Vanderbilt University, Nashville, TN 37203, United States; Vanderbilt University School of Medicine, Department of Health Policy, Vanderbilt University, Nashville, TN 37203, United States; Vanderbilt-Ingram Cancer Center, Nashville, TN 37232, United States; Vanderbilt University School of Medicine, Department of Health Policy, Vanderbilt University, Nashville, TN 37203, United States; Vanderbilt University School of Medicine, Department of Health Policy, Vanderbilt University, Nashville, TN 37203, United States; Geriatric Research, Education, and Clinical Center (GRECC), VA Tennessee Valley Healthcare System, Nashville, TN, United States; Vanderbilt University School of Medicine, Department of Health Policy, Vanderbilt University, Nashville, TN 37203, United States

**Keywords:** Medicare Advantage, cancer

## Abstract

**Introduction:**

Beneficiaries enrolled in Medicare Advantage (MA) and newly diagnosed with cancer may be incentivized to switch coverage, particularly if their MA plan restricts their access to cancer care. Beginning January 2019, the deadline to disenroll from an MA plan changed from February 14 to March 31 and, for the first time, beneficiaries could switch to a different MA plan as opposed to having to enter traditional Medicare.

**Methods:**

We used 2016–2019 Surveillance, Epidemiology, and End Results (SEER) Medicare data to conduct a difference-in-differences analysis, estimating the effect of this new policy on rates of MA plan switching 1 month and 2 months after diagnosis.

**Results:**

For beneficiaries diagnosed with cancer in March, the policy was associated with a modest increase in both overall rates of switching and in switching to a new MA plan 1 month after diagnosis. Results indicated that the policy had a modest positive effect on changes in Medicare coverage for enrollees diagnosed in January when outcomes were measured 2 months after diagnosis.

**Conclusion:**

Relaxing enrollment rules for MA enrollees may not exacerbate adverse selection into traditional Medicare, given this evidence that beneficiaries with high-cost new diagnoses rarely exercised the option to change their MA plan.

## Introduction

Upon becoming eligible for Medicare, beneficiaries can choose from 1 of 2 coverage pathways. Traditional Medicare (TM) provides few restrictions on choice of provider, but beneficiaries must contend with potentially higher out-of-pocket costs. Medicare Advantage (MA) plans require little to no premium and can provide a more comprehensive insurance benefit, including lower out-of-pocket costs for in-network care and supplemental benefits that are not available in TM (eg, vision, hearing, and dental coverage). However, in exchange for these perks, beneficiaries face utilization management tools (eg, prior authorization) and network restrictions that may not include their preferred doctors or hospitals. Enrollment in MA has grown substantially, from covering 31% of all Medicare beneficiaries in 2014 to 52% in 2023.^1^

In order to contain health care costs, plans use strategies such as prior authorization, limitations on the use of services,^2,3^ or narrow networks, which may negatively impact medically complex beneficiaries such as those with cancer^4^ and reduce patient access to specialized care.^2,5,6^ However, since plans are required to cap out-of-pocket spending, MA beneficiaries with cancer receive some financial protections from the high costs of cancer care. Additionally, the supplemental benefits plans can offer such as transportation to medical appointments could be meaningful for this population.

To better understand how the MA program does or does not meet the needs of beneficiaries with cancer, this study examines disenrollment from MA to TM or enrollment in a new MA plan among beneficiaries with new cancer diagnoses. Several studies examined rates of switching among beneficiaries with significant health care needs,^7-11^ such as cancer,^12^ and found higher MA disenrollment rates among those who are more medically complex. Beneficiaries with cancer are particularly vulnerable to the negative consequences of poor network design if they are not able to access specialist care or if they face prior authorizations for care, which has been shown to have a chilling effect on medication adherence.^13^

Beneficiaries have 2 times each year where they can change their coverage choices (outside of special circumstances such as relocating). During Medicare's annual open enrollment period, which takes place each fall from October 15 to December 7, beneficiaries can choose new MA plans or TM. This choice then becomes effective the following January. To limit favorable selection,^14^ beneficiaries are locked into this choice for the year. The Centers for Medicare and Medicaid Services (CMS) also provides MA enrollees with the additional opportunity to leave their MA plan at the beginning of the year. This enrollment window is also known as the Medicare Advantage open enrollment period. In January 2019, CMS extended the deadline to leave an MA plan from February 14 to March 31 and, for the first time, permitted beneficiaries to switch to a different MA plan as opposed to having to enter TM.

This longer enrollment window and ability to remain in MA but move to a new plan may be particularly important for beneficiaries who experience an unexpected health crisis, as it provides them with a grace period to enroll in coverage that better meets their needs. Beneficiaries who have cancer may benefit from this flexibility if the plan they chose prior to their cancer diagnosis does not have specialized cancer centers in network, does not have needed specialists in network, or places restrictions on receiving care. For those considering a switch from their chosen MA plan, enrollment in TM allows these beneficiaries to see any willing provider who accepts Medicare, or they may elect to enroll into a new MA plan that better meets their specific needs. Additionally, the ability to remain in MA (either the current plan or a new MA plan) can be useful for patients who now have a pre-existing condition and can be denied or charged higher premiums for Medigap coverage, which covers out-of-pocket costs for beneficiaries in TM.^15,16^

However, allowing beneficiaries to more easily switch to TM could intensify selection from MA to TM. Furthermore, adding a new option to switch to a new MA plan could mitigate selection into TM but introduce adverse selection between MA plans. Given the implications of this longer disenrollment window in terms of adverse selection and for providing beneficiaries with an opportunity to enroll in coverage that better serves them, we evaluated this policy change on overall rates of switching coverage within 1 and 2 months of a new cancer diagnosis. We also examined the type of switch (switching from MA to TM or switching to a different MA plan) that occurred.

## Data and methods

### Data sources

We used 2016–2019 Surveillance, Epidemiology, and End Results (SEER) data linked to Medicare enrollment files. The National Cancer Institute (NCI) collaborates with CMS to link SEER cancer registry data to Medicare enrollment and claims data. SEER collects data on new cancer diagnoses within the catchment areas of registries supported by NCI. Information on patient sociodemographic characteristics, cancer diagnosis date (month and year), and tumor characteristics (eg, tumor stage) are included in the data (regardless of insurance type). Enrollment data from CMS, also known as the Master Beneficiary Summary File, include information on the beneficiary's enrollment in Medicare (TM and MA).

### Study population

The study population included Medicare beneficiaries enrolled in an MA plan whose first cancer diagnosis occurred in January, March, or April of 2016–2019. We examined beneficiaries diagnosed with breast, colorectal, leukemia, lung, lymphoma, and prostate cancer since these diagnoses represent the most common cancer diagnoses among Medicare beneficiaries. While beneficiaries diagnosed with cancer in the latter half of February would potentially be affected by the policy change, we excluded February diagnoses from the main analysis because these beneficiaries always had the option of disenrolling from MA prior to February 14th. Focusing on January and March diagnoses relative to April diagnoses allowed us to make cleaner comparisons. We also excluded beneficiaries who were dual-eligible for Medicare and Medicaid at the time of diagnosis since these beneficiaries have differing enrollment rules than the Medicare-only population and beneficiaries who died in the month they were diagnosed with cancer.

### Study design

We used a difference-in-differences (DiD) design to examine MA plan changes among individuals diagnosed with cancer before and after the extension of the MA disenrollment deadline, which occurred in 2019. The control group included beneficiaries who received a cancer diagnosis in April of each study year since these beneficiaries were unable to change their plan enrollment except in rare circumstances (eg, the beneficiary moved out of their plan's service area). The first treatment group consisted of beneficiaries diagnosed in January: this group was always able to leave MA for TM, but under the new policy they could also switch to a different MA plan. The second treatment group consisted of beneficiaries diagnosed in March: under the extended deadline for disenrollment, these beneficiaries gained the option to leave MA for TM or change MA plans.

### Study measures

The main outcome was any disenrollment from a beneficiary's MA plan in the month following a new diagnosis. We also examined beneficiaries’ subsequent coverage choice: MA program exit (ie, disenrollment from their MA plan to TM) or MA plan switch (ie, switching MA plans). During the MA open enrollment period, beneficiaries’ enrollment changes are effective on the first day of the subsequent month. For example, a beneficiary who selects a new plan on January 10th will be enrolled in that plan on February 1st.

Regression specifications adjusted for beneficiary sociodemographic and clinical characteristics: age, race/ethnicity (eg, Black, White, Asian, Hispanic, Native American, other, and unknown), sex, original reason for Medicare entitlement (eg, old age and survivor's insurance, disability insurance benefits, and end-stage renal disease [ESRD]/both ESRD and disability insurance benefits), cancer stage (eg, localized, regional, distant, and unknown), cancer type (eg, breast, colorectal, leukemia, lung, lymphoma, or prostate cancer), year and county fixed effects, and total months of previous MA enrollment the year before the beneficiary's diagnosis.

### Analytic approach

We compared characteristics of beneficiaries in our sample using standardized mean differences. We considered covariance imbalanced if the absolute value of the standardized mean difference was greater than 0.20.^17^ We assessed the validity of the DiD design by visually inspecting plots of the unadjusted outcomes and formally testing whether there were pre-period differences in changes in outcomes between the treated and control groups (see event study graphs in Appendix Figures S3 and S4 and S8). We used linear probability models for all outcomes, adjusting for patient sociodemographic and clinical characteristics, and included year and county fixed effects, and robust standard errors.

### Sensitivity analysis

We performed several sensitivity analyses to test the robustness of the results in the main analysis. First, because beneficiaries may have not immediately decided to change their coverage after being diagnosed and because the diagnosis date is imprecise (month and year only), we also measured the outcomes of interest 2 months post-diagnosis for January and April.

Second, there have been recent concerns raised in the DiD literature regarding the conventional approach to statistically testing for pre-period trends, including low power to detect violations of the parallel trends assumption and a desire to conduct the analysis even when parallel trends appear to be violated since the findings may still be informative for policymakers.^18^ To address these concerns, we used an approach developed by Rambachan and Roth^18^ to develop upper and lower bounds for DiD estimates. We report these sensitivity analyses in the Appendix (Figures S9–S11).

Third, we stratified analyses by whether the beneficiary was diagnosed in a SEER state that has guaranteed issue for Medigap coverage (Connecticut, Massachusetts, or New York) or in a state without those protections. In these states, plans cannot deny Medigap enrollment to eligible applicants, including beneficiaries with pre-existing conditions.

Last, we focused the analysis on beneficiaries enrolled in a health maintenance organization (HMO) or preferred provider organization (PPO). Analyses were stratified by whether the beneficiary was enrolled in an HMO or PPO to determine whether the policy differentially impacted beneficiaries in plans with more or less restrictive access to out-of-network coverage.

### Limitations

There were several limitations of the analysis. First, our results do not generalize to dual-eligible beneficiaries with Medicare and Medicaid, a population who faces more flexible rules for plan exit. Second, although we aimed to increase the internal validity of our analysis by using a DiD design, our estimates could be biased if there were other changes in Medicare enrollment rules that differentially affected those diagnosed in January and March vs April. To our knowledge, no other policy changes occurred during this time. Third, we only had 1 year of post-period data. The impact of the policy may increase if beneficiaries become more aware of this option over time. Fourth, we cannot observe the exact timing within a month of a beneficiary's decision to change their Medicare coverage or the exact date of their cancer diagnosis. Some beneficiaries may have made enrollment choices prior to being diagnosed with cancer, particularly since patients undergo a series of diagnostic testing in the lead-up to receiving their diagnosis. The sensitivity analyses that looked at enrollment choices over a longer time window provide greater assurance that the disenrollment choice was likely subsequent to the patient's diagnosis. Last, when we examined how sensitive our results were to pre-policy violations of the parallel trends assumption, we found that our results for the March diagnosis group may be insignificant in the presence of small pre-trend differences; therefore, these results should be interpreted with caution.

## Results

### Cohort characteristics

The study sample included 48 266 MA members newly diagnosed with cancer (mean age [SD]: 73.5 [7.5] years; 47.8% female). When we compared beneficiaries in the treatment group with beneficiaries in the control group using standardized mean differences, we found that these groups were not different based on observable characteristics (Table 1).

**Table 1.** Characteristics of Medicare Advantage enrollees with cancer diagnoses, 2016–2019.

	Beneficiaries diagnosed in January and March	Beneficiaries diagnosed in April	Standardized mean difference
Sample size, *n*	32 639	15 627	
Age (SD), y	73.5 (7.5)	73.6 (7.5)	0.014
Sex, %			
Male	52.4%	51.8%	0.013
Female	47.6%	48.2%	−0.013
Race/ethnicity, %			
Black	12.3%	12.2%	0.002
White	70.3%	70.6%	−0.007
Asian	4.4%	4.4%	−0.001
Hispanic	10.4%	10.2%	0.005
Other/unknown/Native American	4.9%	4.7%	0.010
Reason for Medicare entitlement, %			
OASI	85.9%	85.6%	0.008
DIB, ESRD, both DIB and ESRD	14.1%	14.4%	−0.008
Cancer stage, %			
Localized	50.9%	51.6%	−0.013
Regional	18.4%	19.0%	−0.013
Distant	23.7%	23.3%	0.009
Unknown	6.9%	6.2%	0.031
Cancer type, %			
Breast	25.6%	25.6%	0.001
Colorectal	13.3%	13.6%	−0.009
Leukemia	2.8%	2.6%	0.012
Lung	22.0%	22.4%	−0.011
Lymphoma	6.9%	7.1%	−0.009
Prostate	29.4%	28.6%	0.016
Previous months of MA coverage (SD)	10.9 (3.2%)	10.8 (3.3%)	0.015

Source: Authors' analysis of Surveillance, Epidemiology, and End Results (SEER)–Medicare data, 2016–2019. The following states were included in our analysis: California, Connecticut, Georgia, Hawaii, Idaho, Iowa, Kentucky, Louisiana, Massachusetts, New Jersey, New Mexico, Detroit, Texas, Utah, Seattle (Puget Sound). Previous MA coverage months count the months of MA enrollment the year before diagnosis. Imbalance in covariates defined as absolute value greater than 0.20.

Abbreviations: DIB, disability insurance benefit; ESRD, end-stage renal disease; MA, Medicare Advantage; OASI, Old Age and Survivor's Insurance.

### Main analysis: switching 1 month post-diagnosis

**Figure 1.**
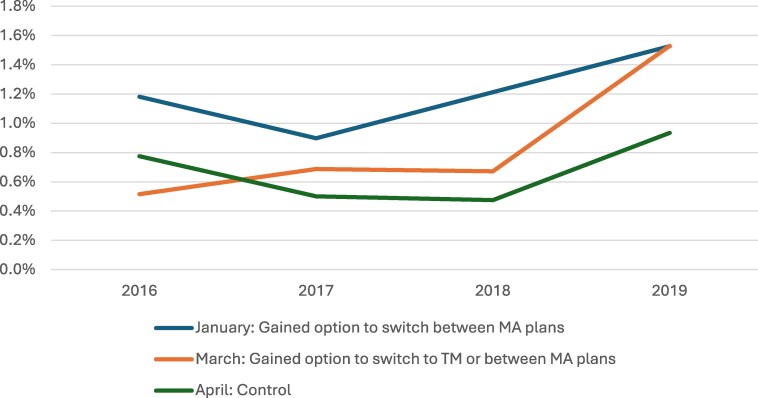
Unadjusted rates of any plan disenrollment by month of diagnosis, 2016–2019. Source: Authors' analysis of SEER-Medicare data, 2016–2019. Outcomes were measured on month post-diagnosis. Abbreviations: MA, Medicare Advantage; SEER, Surveillance, Epidemiology, and End Results; TM, traditional Medicare.

As expected, before the policy was implemented, beneficiaries diagnosed with cancer in January (1.1%) had the highest rates of any switch compared with those diagnosed in March (0.63%) or April (0.58%). Plots of unadjusted outcomes (Figure 1), event study plots, and pre-trend testing (Appendix Figures S3 and S4) indicated that outcomes did not vary by diagnosis month prior to the policy change.

For beneficiaries diagnosed with cancer in January, we found that the policy had no effect on any of the measured outcomes (0.10 percentage point [pp] [95% CI: −0.40, 0.60; *P* = .69] for overall rates of switching; −0.21 pp [95% CI: −0.59, 0.16; *P* = .27] for switching from MA to TM; and 0.31 pp [95% CI: −0.02, 0.64; *P* = .07] for switching to a new MA plan) (Table 2).

**Table 2.** Changes in Medicare enrollment 1 month after cancer diagnosis due to the extended Medicare Advantage open enrollment period, 2016–2019.

	Pre-period mean (2016–2018)			Difference-in-differences estimates			
				January compared with April		March compared with April	
	January	March	April	Coeff (95% CI)	*P*	Coeff (95% CI)	*P*
Any plan disenrollment	1.1%	0.63%	0.58%	0.10 (−0.40, 0.60)	.69	0.66 (0.16, 1.2)	.01
Disenrollment from MA to TM	0.81%	0.33%	0.32%	−0.21 (−0.59, 0.16)	.26	0.22 (−0.14, 0.59)	.22
Switch old MA plan to new MA plan	0.29%	0.30%	0.26%	0.32 (−0.02, 0.64)	.06	0.44 (0.09, 0.79)	.014
Observations				48 266			

Source: Authors' analysis of Surveillance, Epidemiology, and End Results (SEER)–Medicare data, 2016-2019. Beneficiary-level of analysis. Outcomes measured 1 month post–cancer diagnosis. Results from linear probability models with robust standard errors and adjusted for age, race/ethnicity, sex, original reason for Medicare entitlement, cancer stage, cancer type, year and county fixed effects, and total months of previous MA enrollment the year before the beneficiary's diagnosis. Beneficiaries diagnosed in January have always been able to make coverage changes but can now choose to remain in MA and choose a new MA plan. Beneficiaries diagnosed in March are now able to make coverage redeterminations and utilize the option to exit MA for TM or choose a new MA plan.

Abbreviations: Coeff, coefficient; MA, Medicare Advantage; TM, traditional Medicare.

For beneficiaries diagnosed with cancer in March, the policy was associated with an increase in both overall rates of switching and in switching to a new MA plan. The March diagnosis group was 0.66 pp (95% CI: 0.16, 1.16; *P* = .01) more likely to switch overall and 0.44 pp (95% CI: 0.09, 0.79; *P* = .015) more likely to move to a new MA plan compared with the April diagnosis group in the post-period (Table 2). This represented a 105% and 147% relative increase from the pre-period March intervention group's mean, respectively.

### Sensitivity analysis

#### Switching 2 months post-diagnosis

In the first set of sensitivity analyses where we examined outcomes at 2 months post-diagnosis, pre-period rates of switching were higher than in the main analysis, with similar trends for treatment and control groups (Appendix Figure S8). From 2016 to 2018, the overall rate of switching was 5.8% among those diagnosed with cancer in January and 5.5% among those diagnosed in April. When we disaggregated by the type of switch for the January intervention group in the pre-period, rates of disenrolling from MA to TM were 5.3% and the rate of switching to a new MA plan was 0.50%. For the control group, the switch rates were 5.0% and 0.47%, respectively (Table 3).

**Table 3.** Changes in Medicare enrollment 2 months after cancer diagnosis due to the extended Medicare Advantage open enrollment period, 2016–2019.

	Pre-period mean (2016–2018)		Difference-in-differences estimates, January compared with April	
	January	April	Coeff (95% CI)	*P*
Any plan disenrollment	5.8%	5.5%	1.28 (0.20, 2.35)	.02
Disenrollment from MA to TM	5.3%	5.0%	0.60 (−0.40, 1.60)	.24
Switch old MA plan to new MA plan	0.50%	0.47%	0.67 (0.25, 1.09)	.002
Observations			31 785	

Source: Authors' analysis of Surveillance, Epidemiology, and End Results (SEER)–Medicare data, 2016–2019. Outcomes measured 2 months post–cancer diagnosis. Results from linear probability models with robust standard errors and adjusted for age, race/ethnicity, sex, original reason for Medicare entitlement, cancer stage, cancer type, year and county fixed effects, and total months of previous MA enrollment the year before the beneficiary's diagnosis. Beneficiaries diagnosed in January have always been able to make coverage changes but can now choose to remain in MA and choose a new MA plan.

Abbreviations: Coeff, coefficient; MA, Medicare Advantage; TM, traditional Medicare.

The policy had a significant effect on 2 out of the 3 outcomes: a 1.27 pp (95% CI: 0.20, 2.35; *P* = .02) increase in the likelihood of changing coverage overall and a 0.67 pp (95% CI: 0.25, 1.09; *P* = .002) increase in switching to a new MA plan relative to the control group in the post-period (Table 3). This represented a 22% and 134% relative increase, respectively, in the outcome from the treated group's pre-period mean.

#### Honest DiD

Although the event study plots reported in the Appendix (Figures S3, S4, and S8) section of the article do not reveal violations of the parallel trend assumption, recent literature has raised concerns that these tests may be underpowered to detect violations of the assumption. Given this concern, we conducted a set of sensitivity analyses bounding the estimates (Appendix Figures S9–S11). For example, the significant increase in 1-month switching rates among beneficiaries diagnosed in March was robust to allowing violations of the parallel trends assumption that are no more than 0.5 as large as the maximum pre-treatment period violation (Appendix Figure S10).

#### Medigap protection vs non-Medigap protection states

Stratifying by whether the beneficiary was diagnosed in a state with or without Medigap-guaranteed issue protections indicated the policy was associated with lower disenrollment to TM for beneficiaries diagnosed in January and in a non-Medigap protection state (−0.51 pp; 95% CI: −0.85, −0.17; *P* = .003) and a marginally nonsignificant increase in choosing a new MA plan for these enrollees (0.37 pp; 95% CI: −0.01, 0.74; *P* = .05). Beneficiaries in Medigap protection states and diagnosed in March had an increased probability of any switch occurring (1.78 pp; 95% CI: 0.31, 3.24; *P* = .02) (Appendix Table S1).

#### PPO vs HMO

There were largely no differences in outcomes when we stratified by HMO vs PPO. However, there was a modest positive effect on beneficiaries enrolled in PPOs and diagnosed in March (0.58 pp; 95% CI: 0.10, 1.06) (Appendix Table S2).

## Discussion

In this study examining the effects of a longer disenrollment period and the ability of MA enrollees to switch into either TM or a new MA plan, we found that the policy had no effect on rates of switching within 1 month of diagnosis for beneficiaries diagnosed with cancer in January.

However, the policy was associated with modest increases in overall rates of switching and switching to a new MA plan for those diagnosed with cancer in March relative to the control group. When we examined switching outcomes over 2 months for beneficiaries diagnosed in January, we found that the policy increased overall rates of switching and rates of switching to a new MA plan.

Stratifying analyses by whether the beneficiary was diagnosed in a state with or without guaranteed issue revealed a decline in exits to TM for beneficiaries diagnosed in January in a non-Medigap state. There was a modest positive increase in beneficiaries utilizing the option to remain in MA and switch to a new plan, although this result was only marginally nonsignificant. This could indicate that the beneficiaries in states without access to protections from medical underwriting in the Medigap market may be taking advantage of the opportunity to avoid financial implications of being enrolled in TM with no supplemental coverage.

While it appears that the policy had no effect on rates of exiting MA for TM for either treatment group, the policy consistently led to increases in enrollment in a new MA plan switching, albeit from a low base rate. These results are consistent with a prior study that evaluated this policy among beneficiaries using skilled nursing facility and home health services.^19^ This preference to stay within the MA program may indicate that beneficiaries value aspects of MA coverage such as low premiums, out-of-pocket maximums, or supplemental benefits. Additionally, aspects of the TM program likely make it a challenging option financially for patients newly diagnosed with a serious illness such as cancer. Under the Part B program in TM, beneficiaries are responsible for 20% coinsurance on all outpatient services with no cap on spending. Many beneficiaries^20^ obtain Medigap to cover TM's cost-sharing. However, Medigap plans can medically underwrite beneficiaries or deny them coverage. Eight states protect beneficiaries from these practices, which are associated with higher rates of staying enrolled in TM among beneficiaries who switch from MA to TM.^15^ Beneficiaries with cancer are especially vulnerable to TM's cost-sharing structure given the high costs of cancer care, which may contribute to their preference to remain in MA and not enter TM.

However, our results reveal only modest increases in switching to a new MA plan, which could more generally point to “stickiness” in coverage choices.^21^ Previous qualitative work has highlighted enrollee hesitance to switch coverage,^22^ and other studies examining Part D enrollment find that beneficiaries do not switch coverage even when better options are available.^23^ Our modest findings could also be a result of a long period of time to establish a cancer diagnosis, as patients wait for referrals and testing prior to a formal diagnosis. Since we included beneficiaries with a confirmed cancer diagnosis in the beginning of the year, it is possible that beneficiaries might have experienced access challenges with their plan during the annual Medicare open enrollment period (October–December) and opted for more comprehensive coverage before their diagnosis was confirmed.

## Conclusion

A new policy that provided a longer disenrollment window for MA enrollees was not associated with increased disenrollment among beneficiaries newly diagnosed with cancer in January. However, beneficiaries diagnosed with cancer in March did experience increased rates of switching overall and used the new ability to remain in MA but choose a new MA plan, albeit moderately. Relaxing enrollment rules for MA enrollees may not exacerbate adverse selection into TM, given that beneficiaries with high-cost new diagnoses rarely exercised the choice to switch coverage.

## Supplementary Material

qxaf131_Supplementary_Data
