# Supplementary material for: The effect of providing Medicare Advantage enrollees diagnosed with cancer additional time to reassess enrollment
Source: Health Aff Sch. 2025 Jun 27;3(7):qxaf131. doi: 10.1093/haschl/qxaf131 (PMC12248418; doi:10.1093/haschl/qxaf131)
Supplement: qxaf131_Supplementary_Data [file qxaf131_supplementary_data.zip › Supplemental Appendix (2).docx]

**Supplemental Appendix**

**Figure 1.** Unadjusted rates of disenrollment from MA plan to TM by month of diagnosis, 2016-2019

**Source:** Author’s analysis of SEER-Medicare data, 2016-2019.

**Note:** Outcomes measured on month post-diagnosis

**Figure 2.** Unadjusted rates of switching MA plans by month of diagnosis, 2016-2019

**Source:** Author’s analysis of SEER-Medicare data, 2016-2019.

**Note:** Outcomes measured on month post-diagnosis

**Figure 3.** Event study estimates for January diagnoses


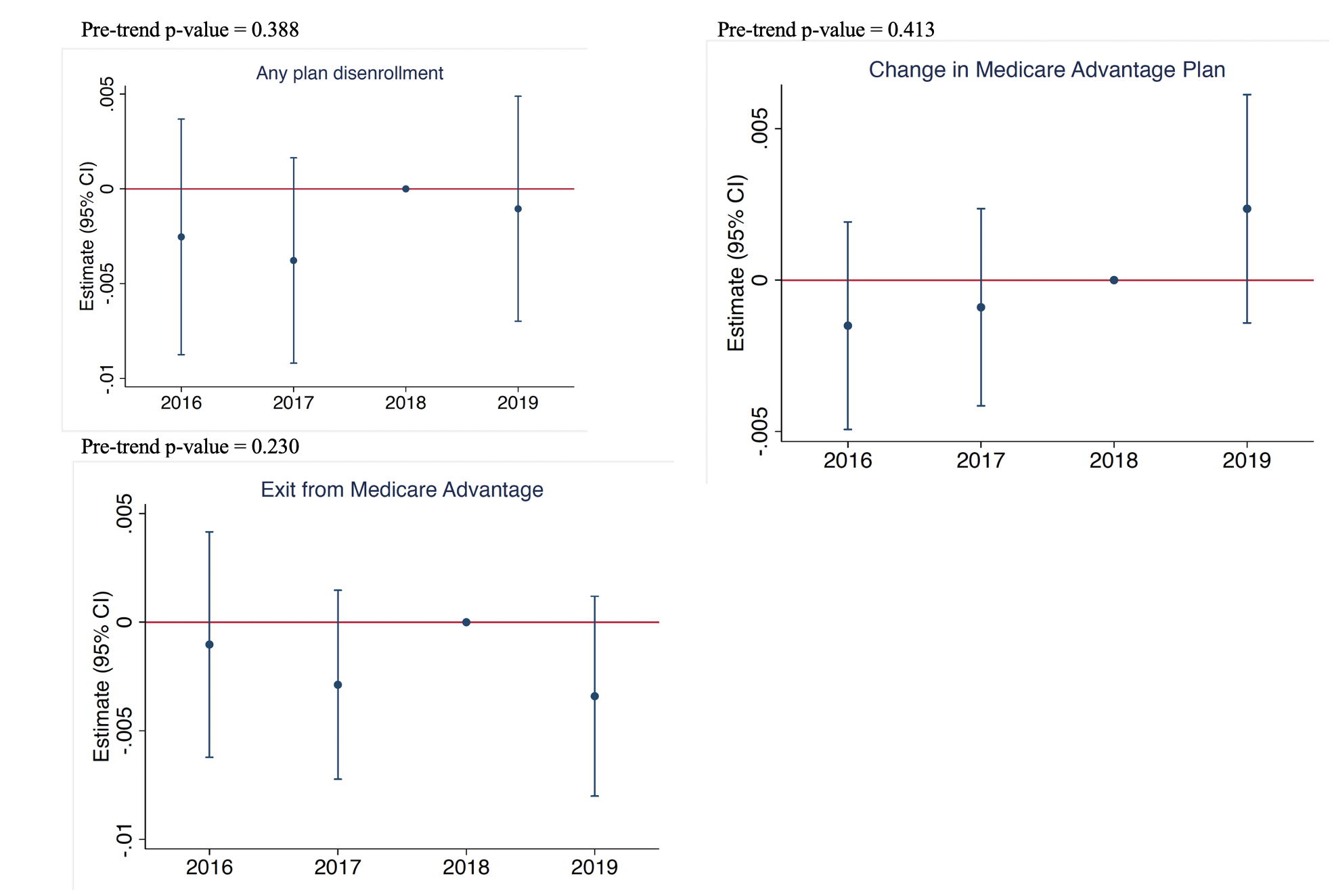


**Source:** Author’s analysis of SEER-Medicare data, 2016-2019

**Note:** Outcomes measured one-month post-diagnosis

**Figure 4.** Event study estimates for March diagnoses


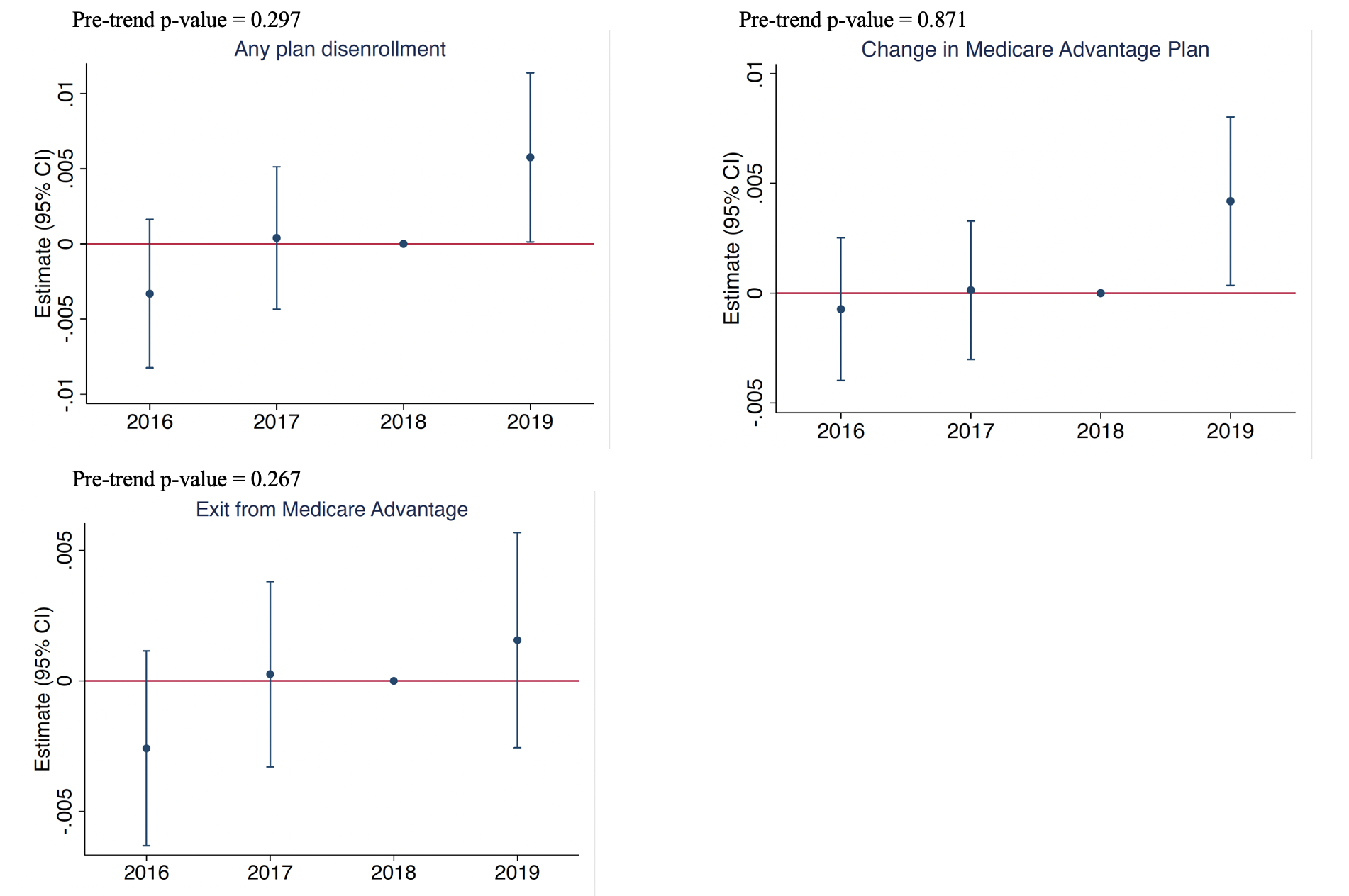


**Source:** Author’s analysis of SEER-Medicare data, 2016-2019

**Note:** Outcomes measured one-month post-diagnosis

**Figure 5.** Unadjusted rates of any plan disenrollment by month of diagnosis, 2016-2019

**Source:** Author’s analysis of SEER-Medicare data, 2016-2019.

**Note:** Outcomes measured two-months post-diagnosis.

**Figure 6.** Unadjusted rates of disenrollment from MA plan to TM by month of diagnosis, 2016-2019

**Source:** Author’s analysis of SEER-Medicare data, 2016-2019.

**Note:** Outcomes measured two-months post-diagnosis

**Figure 7.** Unadjusted rates of switching to new MA plans by month of diagnosis, 2016-2019

**Source:** Author’s analysis of SEER-Medicare data, 2016-2019.

**Note:** Outcomes measured two-months post-diagnosis

**Figure 8.** Event study estimates for January diagnoses

**
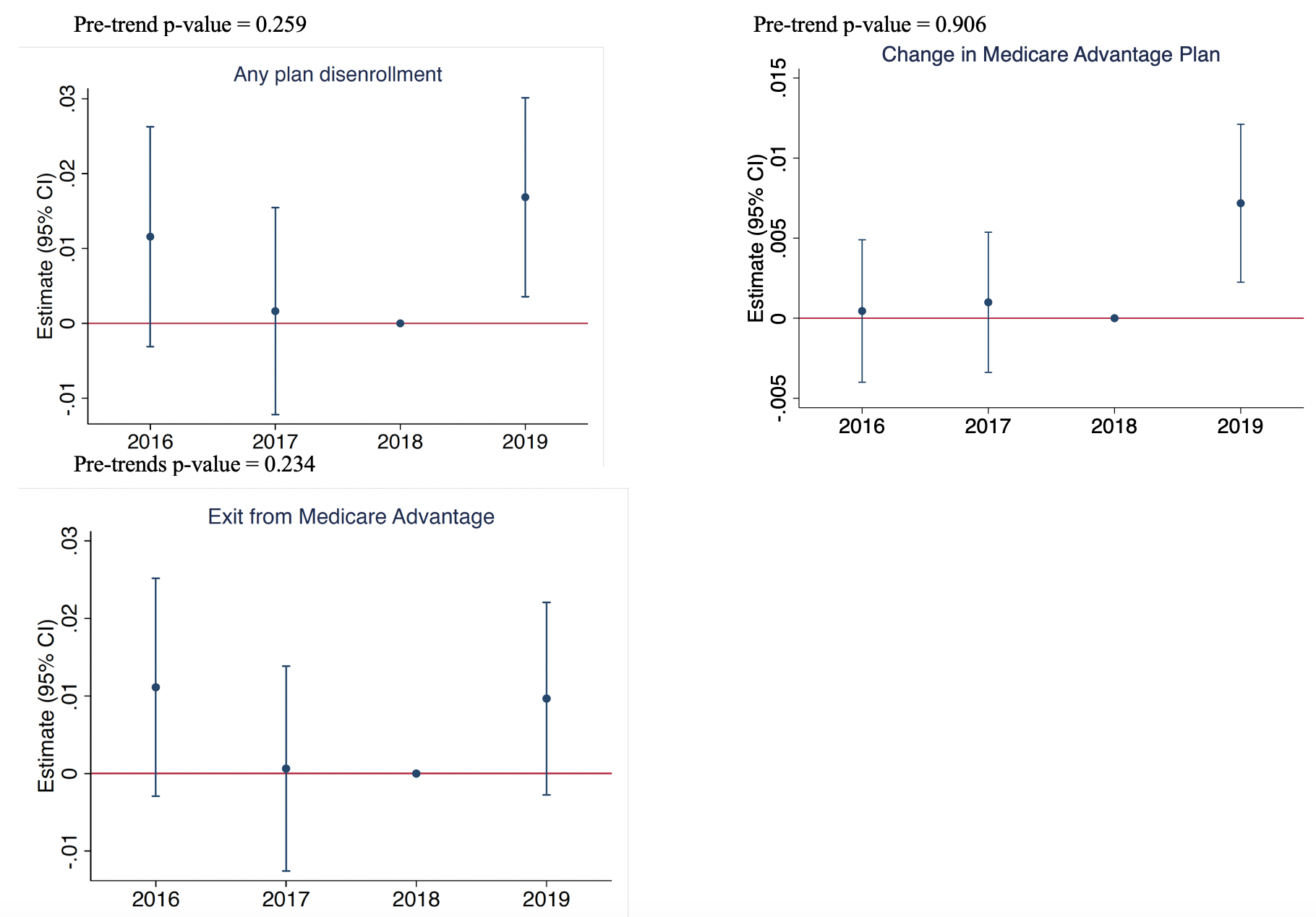
**

**Source:** Author’s analysis of SEER-Medicare data, 2016-2019

**Note:** Outcomes measured two-months post-diagnosis

**Figure 9.** Honest DiD results, January diagnoses

**
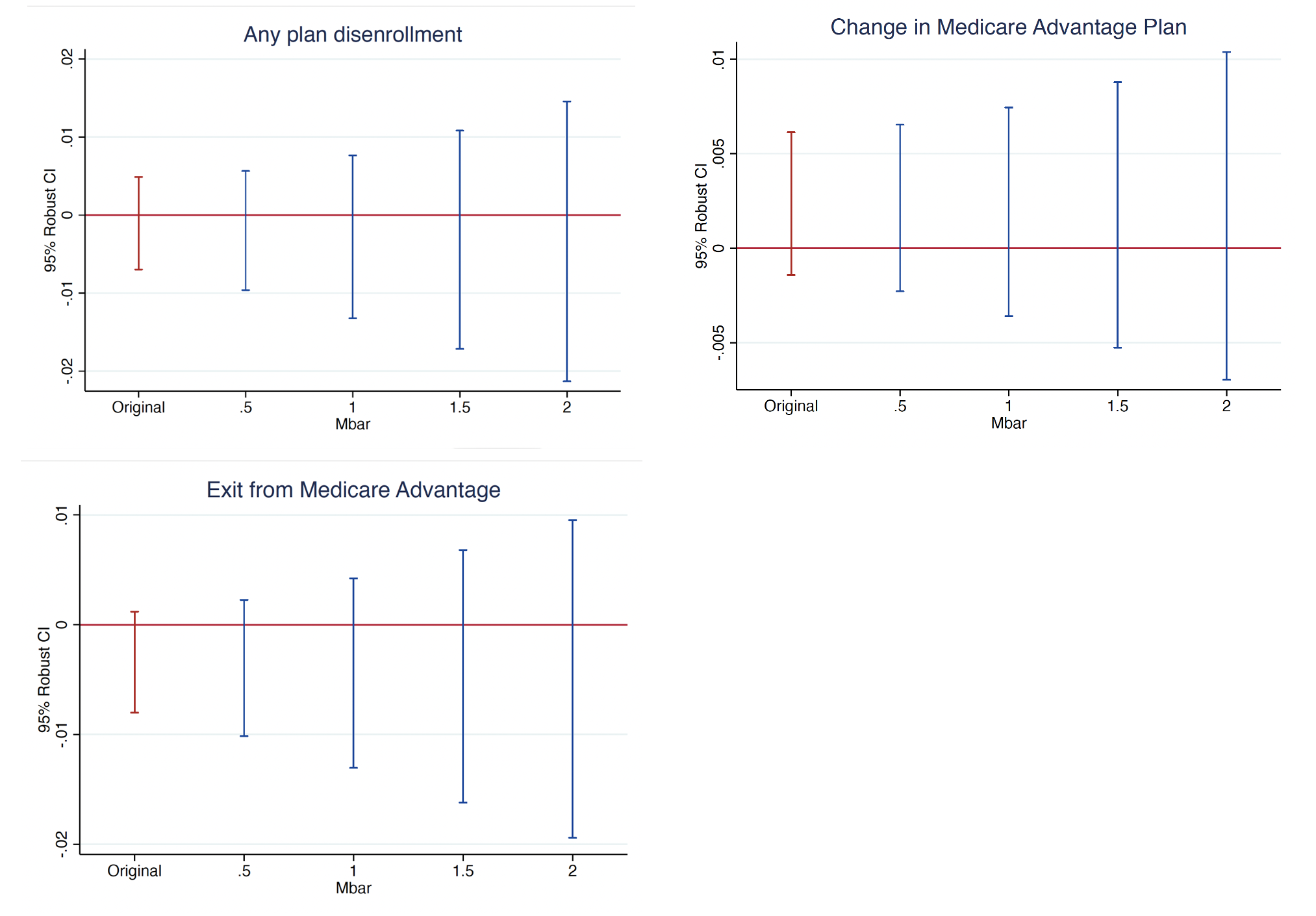
**

**Source:** Author’s analysis of SEER-Medicare data, 2016-2019

**Note:** Outcomes measured one-month post-diagnosis

**Figure 10.** Honest DiD, March diagnoses

**
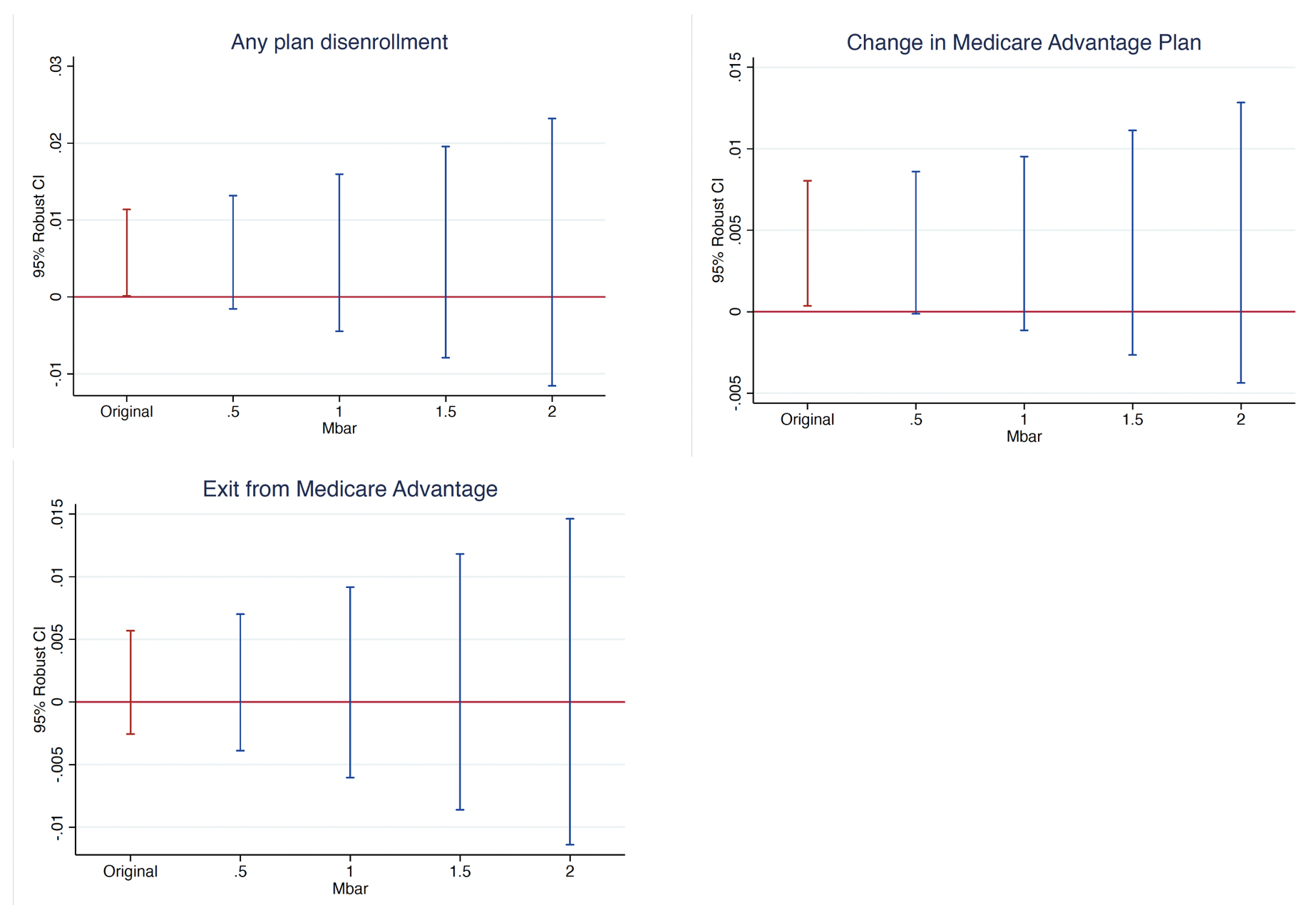
**

**Source:** Author’s analysis of SEER-Medicare data, 2016-2019

**Note:** Outcomes measured one month post diagnosis

**Figure 11.** Honest DiD, January diagnoses

**
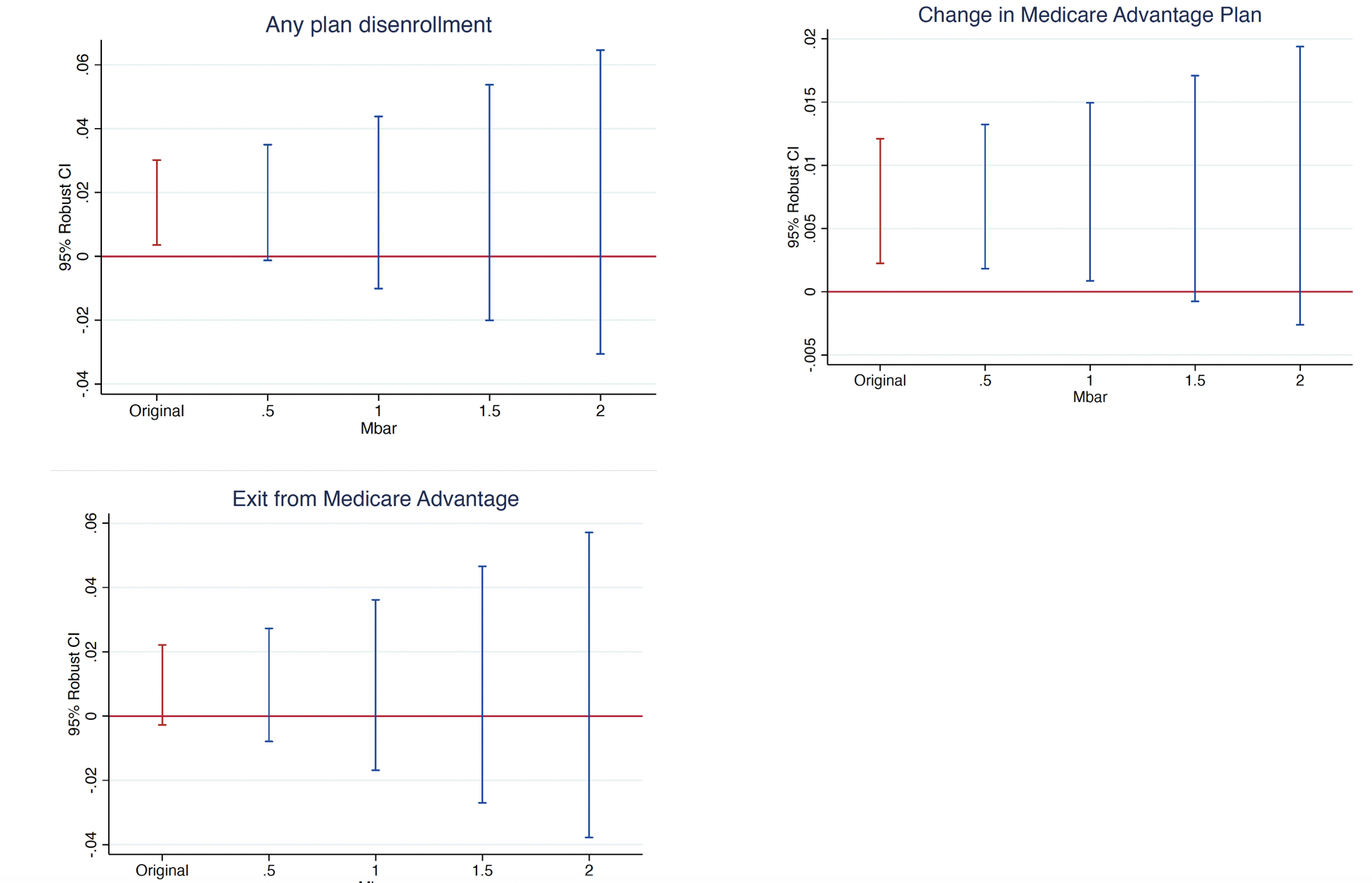
**

**Source:** Author’s analysis of SEER-Medicare data, 2016-2019.

**Note:** Outcomes measured two months post-diagnosis.

**Figure 12.** Event study estimates for January diagnoses, Non-Medigap States


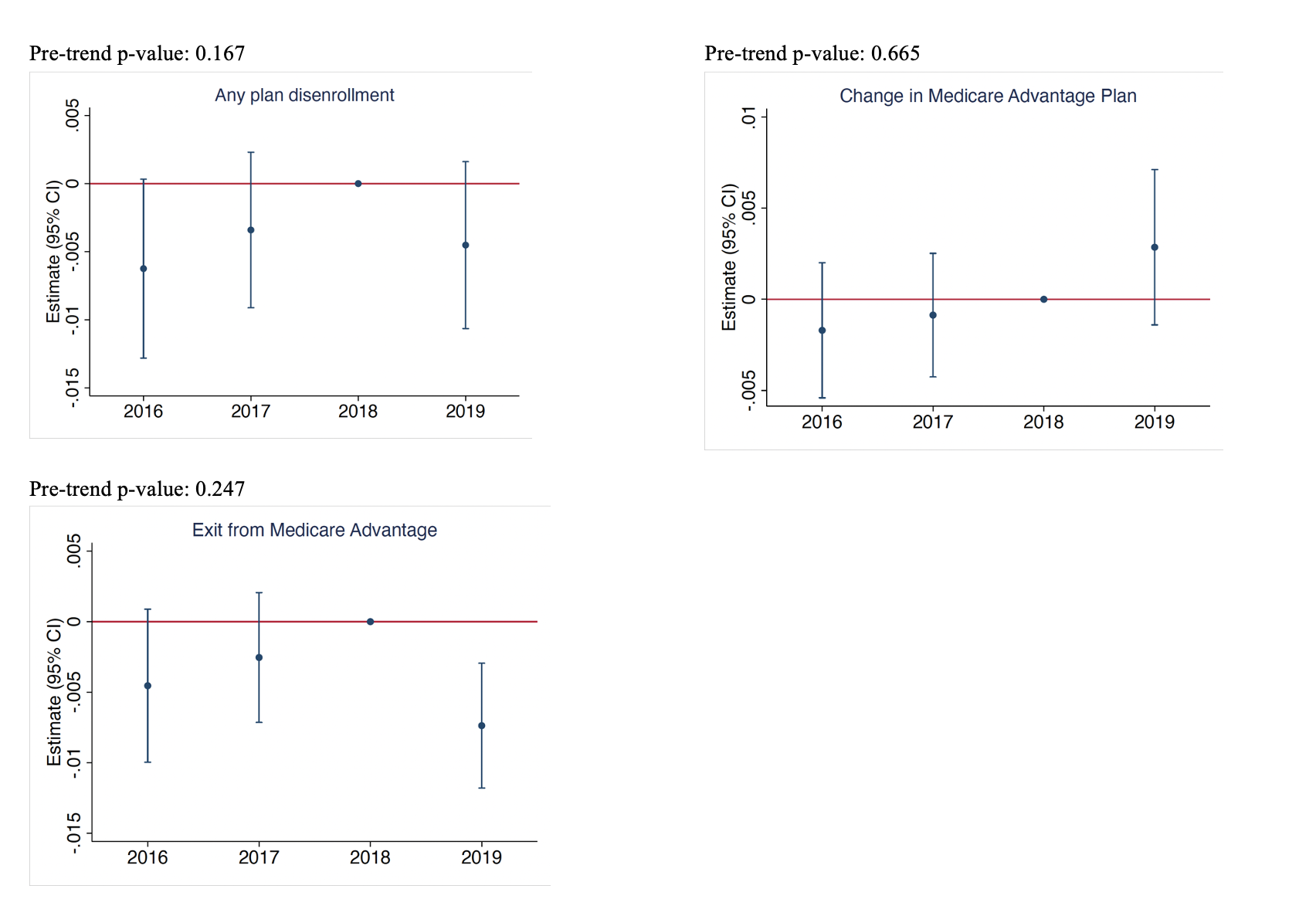


**Source:** Author’s analysis of SEER-Medicare data, 2016-2019.

**Note:** Outcomes measured one-month post-diagnosis.

**Figure 13.** Event study estimates for March diagnoses, Non-Medigap States


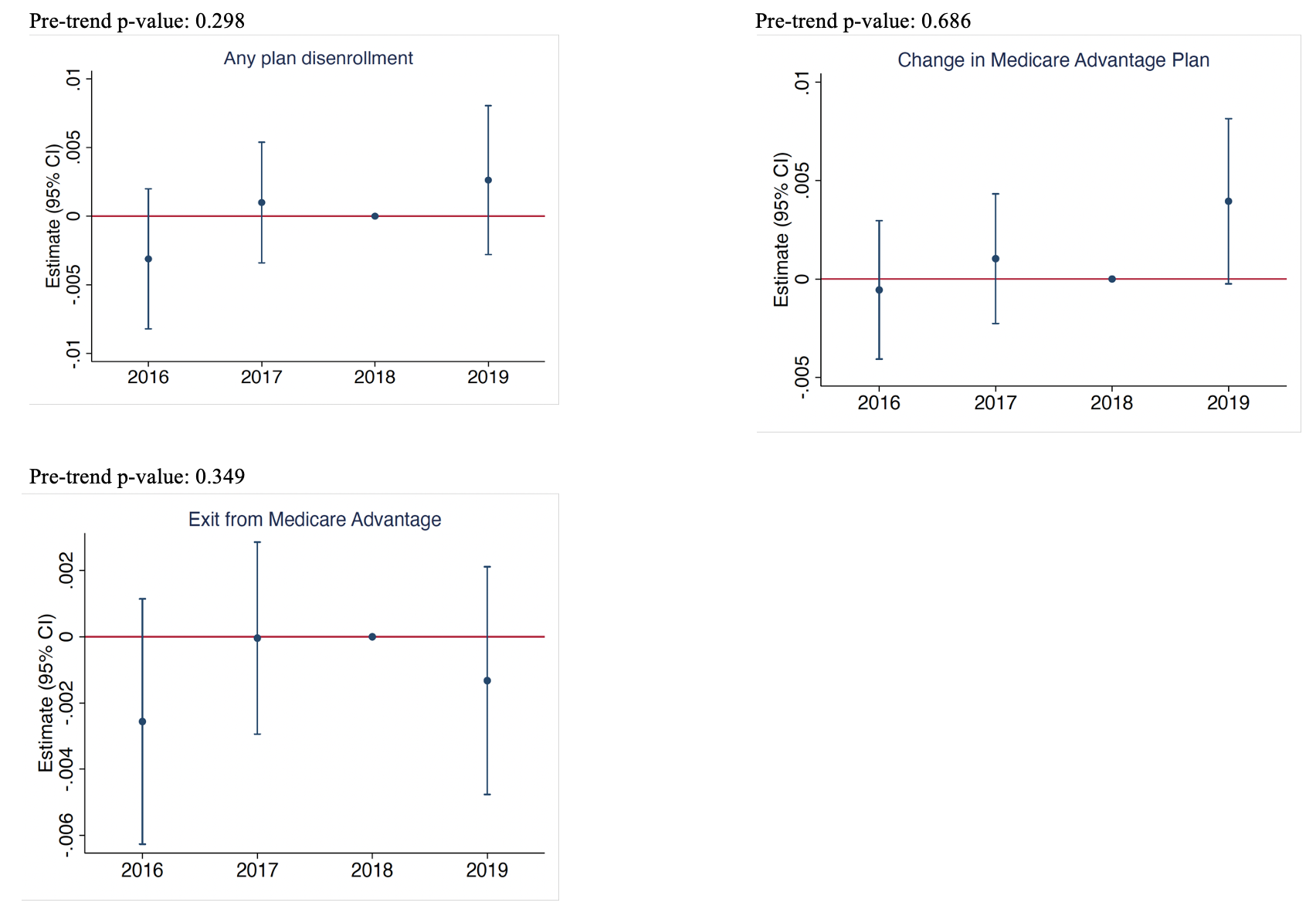


**Source:** Author’s analysis of SEER-Medicare data, 2016-2019.

**Note:** Outcomes measured one-month post-diagnosis.

**Figure 14.** Event study estimates for January diagnoses, Medigap States


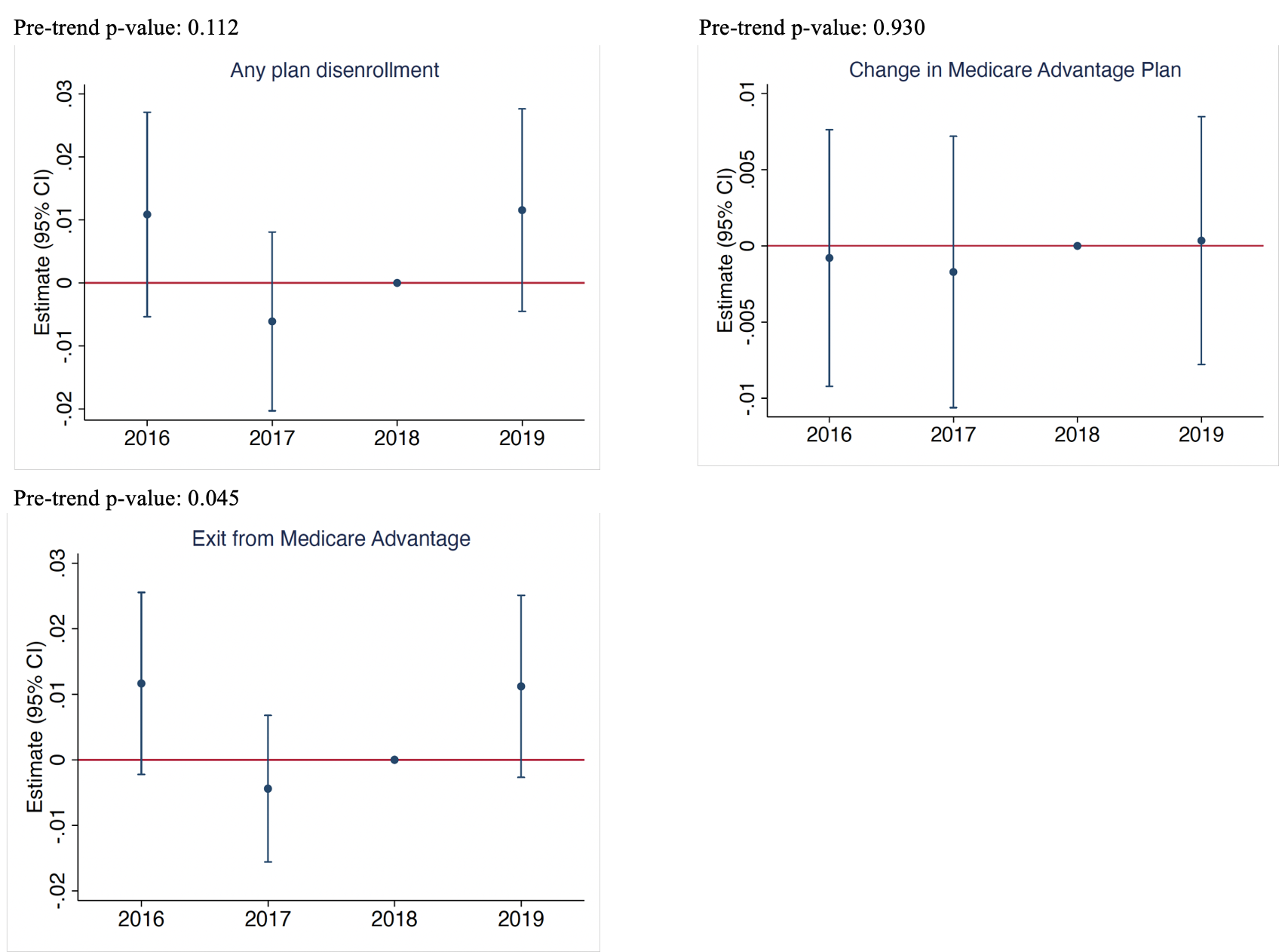


**Source:** Author’s analysis of SEER-Medicare data, 2016-2019.

**Note:** Outcomes measured one-month post-diagnosis.

**Figure 15.** Event study estimates for March diagnoses, Medigap States

**
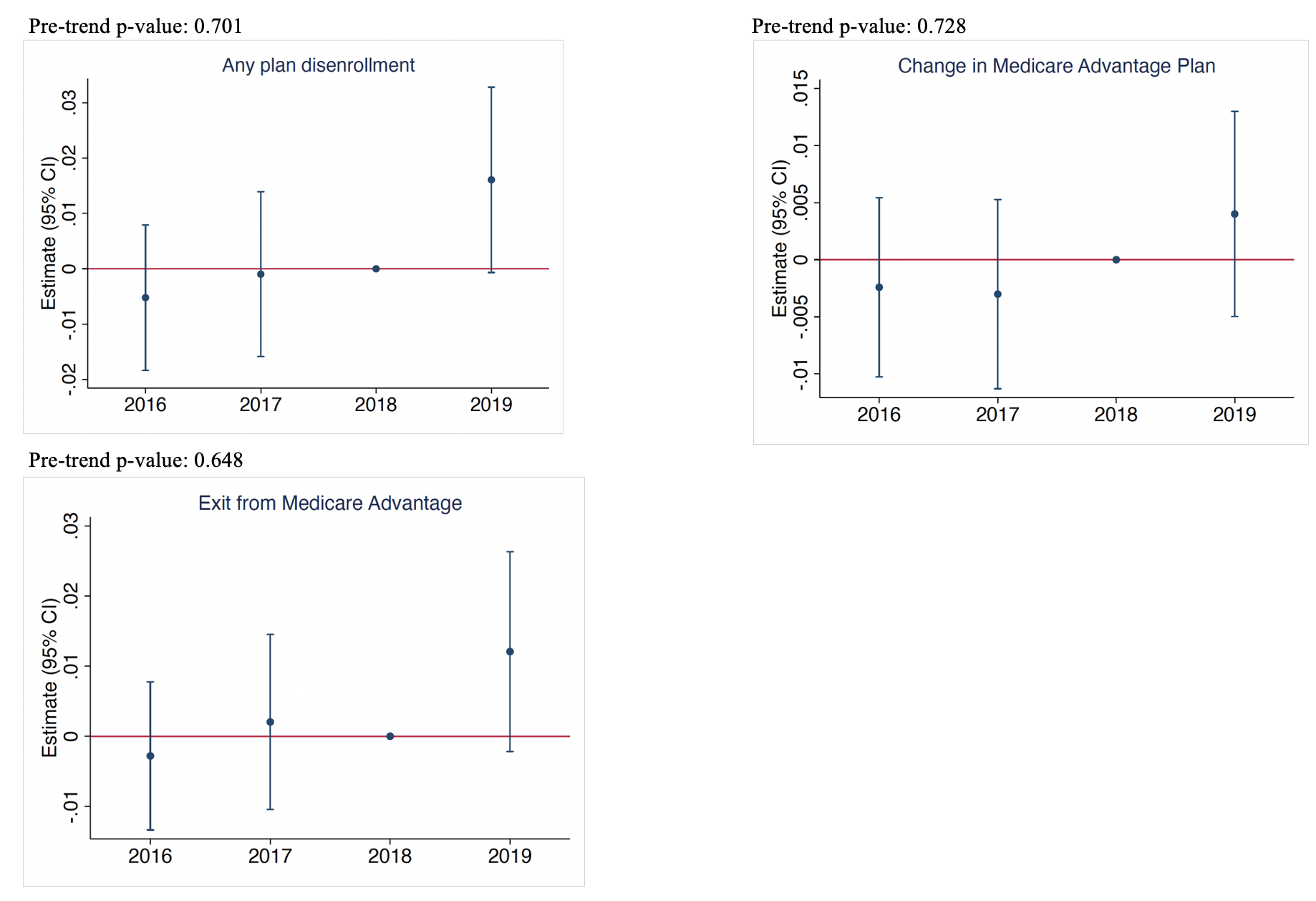
**

**Source:** Author’s analysis of SEER-Medicare data, 2016-2019.

**Note:** Outcomes measured one-month post-diagnosis.

**Table 1.** Changes in Medicare enrollment one month after cancer diagnosis due to the extended Medicare Advantage Open Enrollment Period stratified by states with and without guaranteed issue protections**,** 2016-2019

|  | Non-Medigap States | | | | | | |
| --- | --- | --- | --- | --- | --- | --- | --- |
|  | Pre-period mean  2016-2018 | | | Difference-in-Differences Estimates | | | |
|  |  |  |  | January compared to April | | March compared to April | |
|  | January | March | April | Coeff (95% CI) | p-value | Coeff (95% CI) | p-value |
| Any plan disenrollment | 0.99% | 0.50% | 0.46% | -0.14 (-0.65, 0.36) | 0.58 | 0.32 (-0.17, 0.82) | 0.198 |
| Disenrollment from MA plan | 0.75% | 0.21% | 0.23% | -0.51 (-0.85, -0.17) | 0.003 | -0.05 (-0.36, 0.26) | 0.741 |
| Switch MA plan | 0.24% | 0.28% | 0.23% | 0.37 (-0.01, 0.74) | 0.05 | 0.38 (-0.01, 0.76) | 0.05 |
| Observations |  |  |  | 37,968 | | | |
| Medigap States | | | | | | | |
|  | Pre-period mean  2016-2018 | | | Difference-in-Differences Estimates | | | |
|  |  |  |  | January compared to April | | March compared to April | |
|  | January | March | April | Coeff (95% CI) | p-value | Coeff (95% CI) | p-value |
| Any plan disenrollment | 1.5% | 1.1% | 1.0% | 1.03 (-0.37, 2.42) | 0.15 | 1.78 (0.31, 3.24) | 0.02 |
| Disenrollment from MA plan | 1.0% | 0.8% | 0.6% | NR | 0.13 | 1.20 (-0.02, 2.42) | 0.05 |
| Switch MA plan | 0.5% | 0.4% | 0.4% | 0.11 (-0.61, 0.84) | 0.76 | 0.57 (-0.25, 1.40) | 0.17 |
| Observations |  |  |  | 10,298 | | | |

**Source:** Author’s analysis of SEER-Medicare data, 2016-2019.

**Notes:** Beneficiary-level of analysis. Outcomes measured one-month post cancer diagnosis. Results from linear probability models with robust standard errors and adjusted for age, race/ethnicity, sex, original reason for Medicare entitlement, cancer stage, cancer type, year and county fixed effects, and total months of previous Medicare Advantage (MA) enrollment the year before the beneficiary’s diagnosis. Beneficiaries diagnosed in January have always been able to make coverage changes but can now choose to remain in MA and choose a new MA plan. Beneficiaries diagnosed in March are now able to make coverage redeterminations and utilize the option to exit MA for traditional Medicare or choose a new MA plan. Medigap states included: Connecticut, Massachusetts, and New York.

NR: Denotes pre-trend p-value <0.05.

**Figure 16.** Event study estimates for January diagnoses, Beneficiaries enrolled in HMOs in the month of diagnosis

**
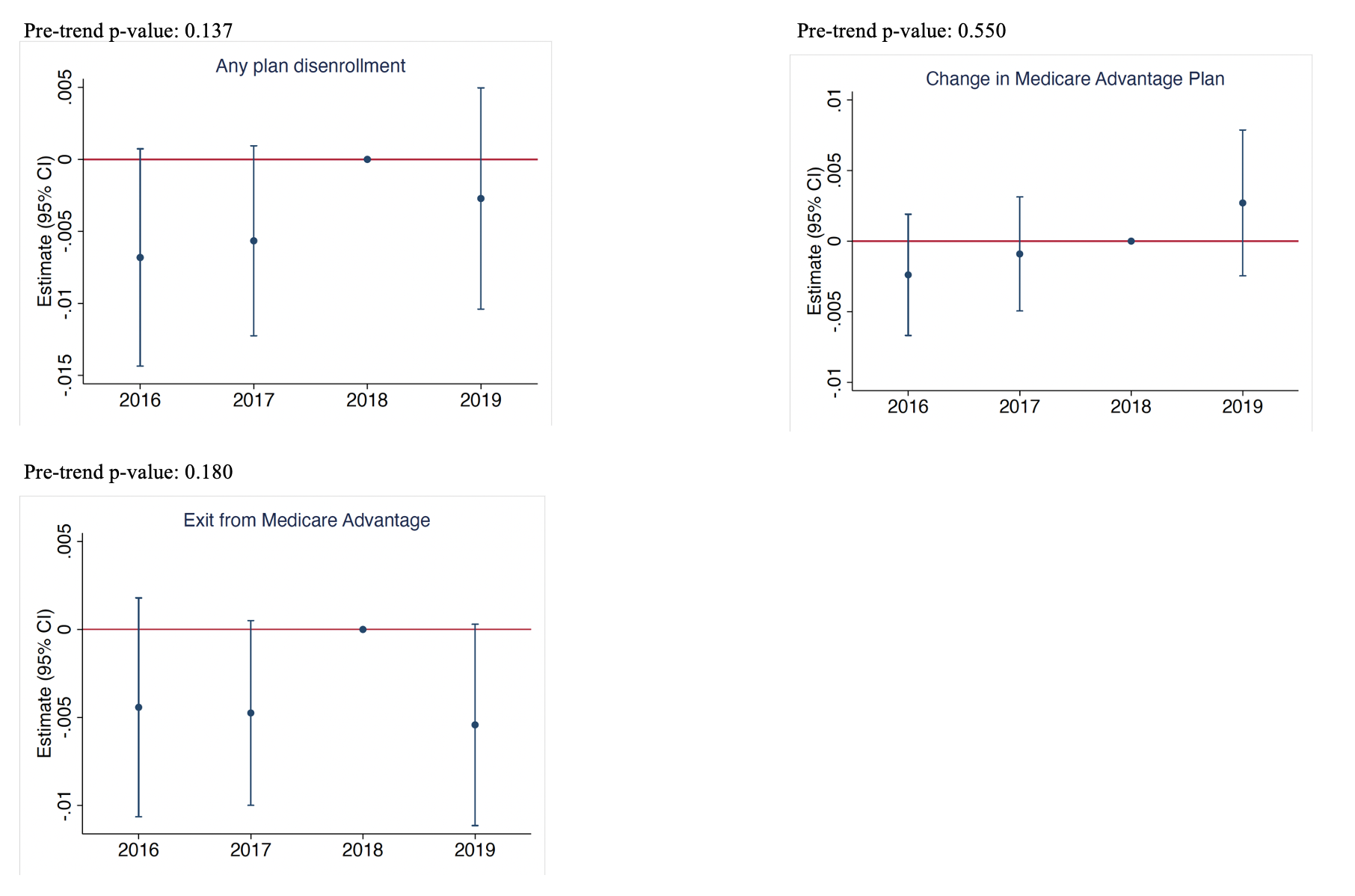
**

**Source:** Author’s analysis of SEER-Medicare data, 2016-2019.

**Note:** Outcomes measured one-month post-diagnosis. Health maintenance organization (HMO).

**Figure 17.** Event study estimates for March diagnoses, Beneficiaries enrolled in HMOs in the month of diagnosis


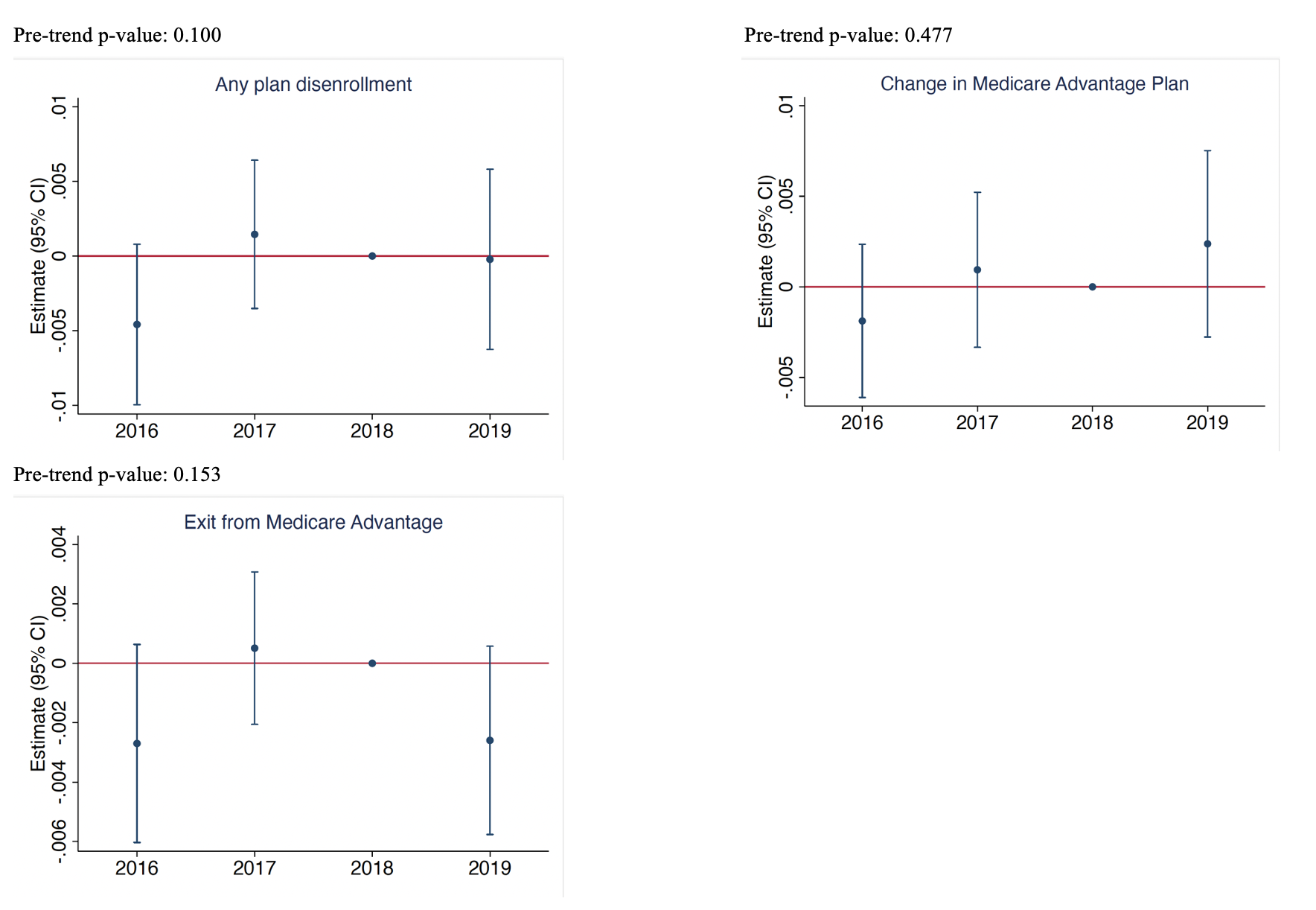


**Source:** Author’s analysis of SEER-Medicare data, 2016-2019.

**Note:** Outcomes measured one-month post-diagnosis. Health maintenance organization (HMO).

**Figure 18.** Event study estimates for January diagnoses, Beneficiaries enrolled in PPOs in the month of diagnosis


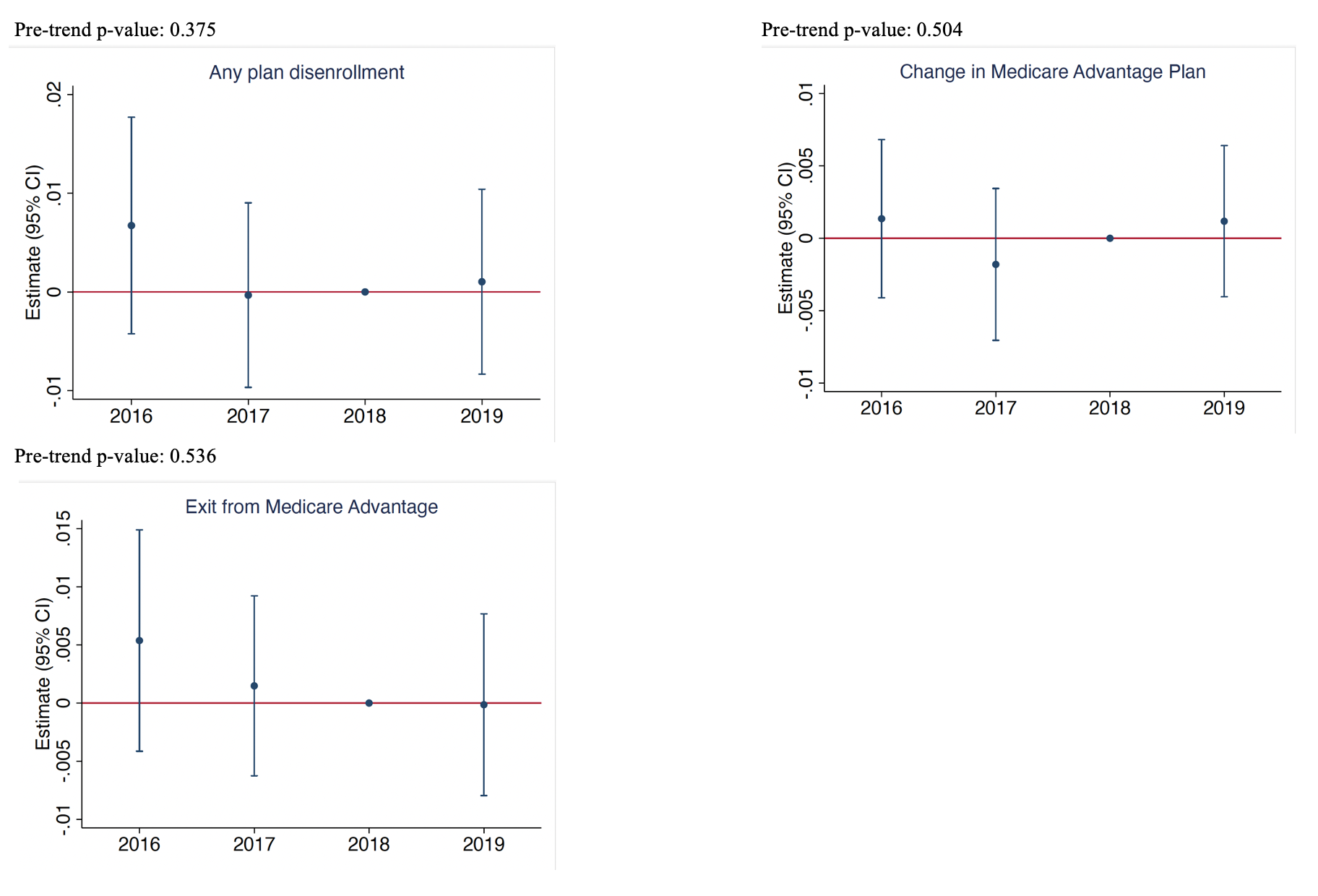


**Source:** Author’s analysis of SEER-Medicare data, 2016-2019.

**Note:** Outcomes measured one-month post-diagnosis. Preferred provider organization (PPO).

**Figure 19.** Event study estimates for March diagnoses, Beneficiaries enrolled in PPOs in the month of diagnosis


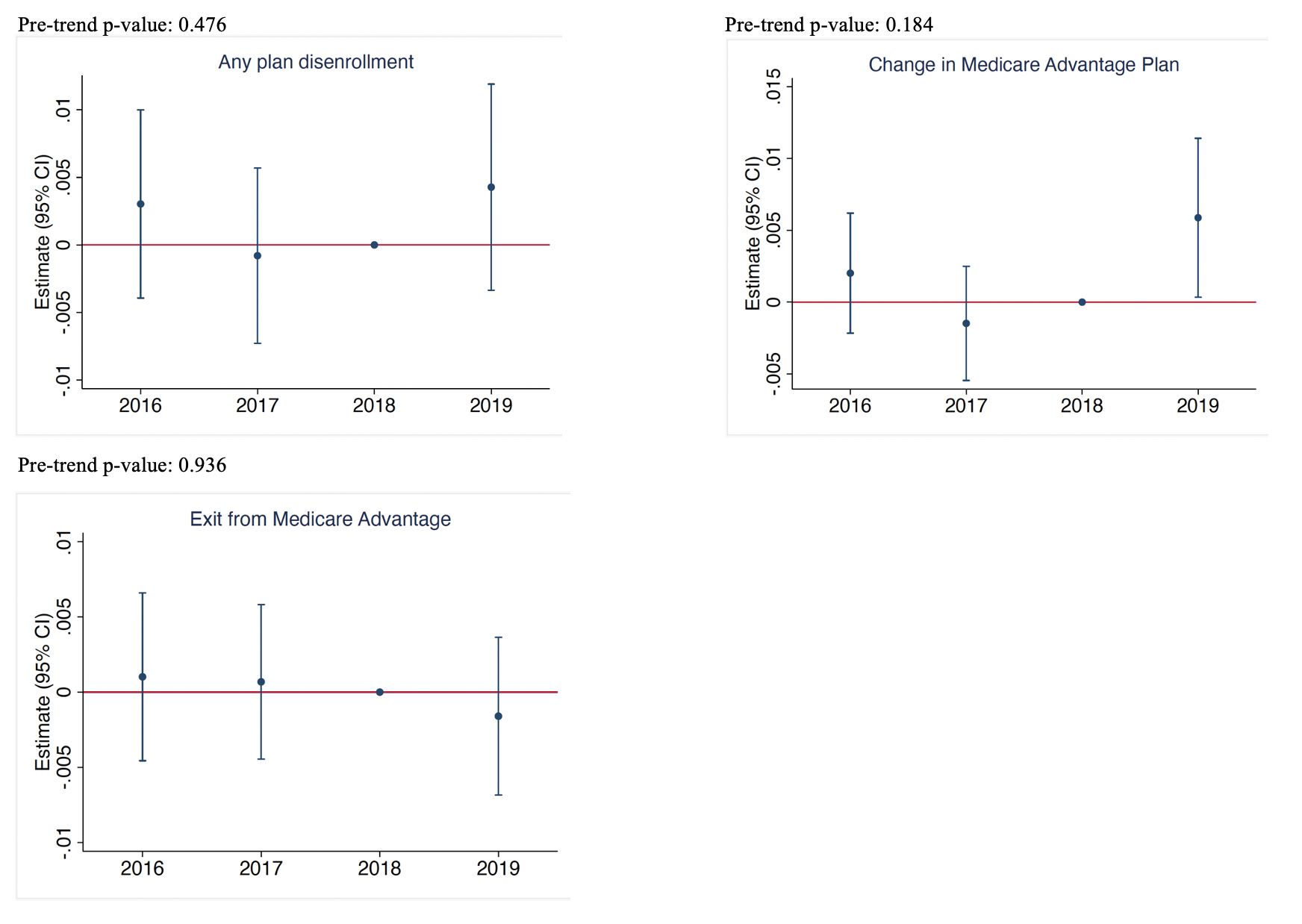


**Source:** Author’s analysis of SEER-Medicare data, 2016-2019.

**Note:** Outcomes measured one-month post-diagnosis. Preferred provider organization (PPO).

**Table 2.** Changes in Medicare enrollment one month after cancer diagnosis due to the extended Medicare Advantage Open Enrollment Period stratified by HMO vs. PPO**,** 2016-2019

| HMO | | | | | | | |
| --- | --- | --- | --- | --- | --- | --- | --- |
|  |  |  |  | Difference-in-Differences Estimates | | | |
|  |  |  |  | January compared to April | | March compared to April | |
|  | January | March | April | Coeff (95% CI) | p-value | Coeff (95% CI) | p-value |
| Any plan disenrollment | 1.06% | 0.39% | 0.59% | 0.14 (-0.51, 0.79) | 0.68 | 0.07 (-0.48, 0.63) | 0.79 |
| Disenrollment from MA plan | 0.78% | 0% | 0.30% | -0.24 (-0.69, 0.21) | 0.30 | -0.19 (-0.48, 0.09) | 0.19 |
| Switch MA plan | 0.29% | 0.39% | 0.30% | 0.38 (-0.09, 0.85) | 0.12 | 0.27 (-0.21, 0.74) | 0.28 |
|  |  |  |  | 30,407 | | | |
| PPO | | | | | | | |
|  |  |  |  | Difference-in-Differences Estimates | | | |
|  |  |  |  | January compared to April | | March compared to April | |
|  | January | March | April | Coeff (95% CI) | p-value | Coeff (95% CI) | p-value |
| Any plan disenrollment | 1.1% | 0.13% | 0.51% | -0.07 (-0.85, 0.70) | 0.86 | 0.37 (-0.27, 1.01) | 0.26 |
| Disenrollment from MA plan | 0.82% | 0.0% | 0.35% | -0.21 (-0.87, 0.44) | 0.53 | -0.21 (-0.63, 0.21) | 0.33 |
| Switch MA plan | 0.28% | 0.13% | 0.16% | 0.14 (-0.28, 0.56) | 0.51 | 0.58 (0.10, 1.06) | 0.02 |
|  |  |  |  | 16,902 | | | |

**Source:** Author’s analysis of SEER-Medicare data, 2016-2019.

**Notes:** Beneficiary-level of analysis. Outcomes measured one-month post cancer diagnosis. Results from linear probability models with robust standard errors and adjusted for age, race/ethnicity, sex, original reason for Medicare entitlement, cancer stage, cancer type, year and county fixed effects, and total months of previous Medicare Advantage (MA) enrollment the year before the beneficiary’s diagnosis. Beneficiaries diagnosed in January have always been able to make coverage changes but can now choose to remain in MA and choose a new MA plan. Beneficiaries diagnosed in March are now able to make coverage redeterminations and utilize the option to exit MA for traditional Medicare or choose a new. Preferred provider organization (PPO), Health maintenance organization (HMO).

**Table 3.** Changes in Medicare enrollment one month after cancer diagnosis due to the extended Medicare Advantage Open Enrollment Period**,** 2016-2019

|  | Any Switch | | Disenrollment from  MA to TM | | Switch old MA plan to  new MA plan | |
| --- | --- | --- | --- | --- | --- | --- |
|  | Coeff (95% CI) | p-value | Coeff (95% CI) | p-value | Coeff (95% CI) | p-value |
| Diagnosis Month |  |  |  |  |  |  |
| April | Ref |  | Ref |  | Ref |  |
| January | 0.49 (0.25, 0.73) | <0.001 | 0.49 (0.30, 0.69) | <0.001 | -0.001 (-0.14, 0.14) | 0.99 |
| March | 0.01 (-0.18, 0.21) | 0.89 | 0.02 (-0.12, 0.17) | 0.75 | -0.01 (-0.14, 0.13) | 0.89 |
| Post (2019) |  |  |  |  |  |  |
| No | Ref |  | Ref |  | Ref |  |
| Yes | 0.27 (-0.06, 0.60) | 0.11 | 0.12 (-0.13, 0.37) | 0.34 | 0.15 (-0.07, 0.36) | 0.19 |
| April*post | Ref |  | Ref |  | Ref |  |
| January*post | 0.10 (-0.40, 0.60) | 0.69 | -0.21 (-0.59, 0.16) | 0.27 | 0.31 (-0.02, 0.64) | 0.07 |
| March*post | 0.66 (0.16, 1.16) | 0.01 | 0.23 (-0.14, 0.59) | 0.22 | 0.44 (0.09, 0.79) | 0.01 |
| Age | -0.02 (-0.03, 0.00) | 0.01 | -0.01 (-0.02, 0.00) | 0.01 | 0.00%  -0.01%  0.00% | 0.41 |
| Sex |  |  |  |  |  |  |
| Female | Ref |  | Ref |  | Ref |  |
| Male | -0.26 (-0.52, 0.01) | 0.06 | -0.13 (-0.34, 0.08) | 0.24 | -0.13 (-0.30, 0.04) | 0.13 |
| Reason for Medicare entitlement |  |  |  |  |  |  |
| OASI | Ref |  | Ref |  | Ref |  |
| DIB, ESRD, Both DIB& ESRD | 0.01 (-0.27, 0.30) | 0.93 | -0.11 (-0.31, 0.10) | 0.32 | 0.12 (-0.08, 0.31) | 0.23 |
| Race/ethnicity |  |  |  |  |  |  |
| Black | Ref |  | Ref |  | Ref |  |
| White | -0.49 (-1.41, 0.44) | 0.30 | -0.70 (-1.57, 0.17) | 0.12 | 0.21 (-0.11, 0.53) | 0.19 |
| Asian | -0.38 (-1.34, 0.58) | 0.44 | -0.84 (-1.73, 0.05) | 0.06 | 0.46 (0.09, 0.82) | 0.01 |
| Hispanic | -0.21 (-1.25, 0.83) | 0.70 | -0.40 (-1.37, 0.56) | 0.41 | 0.19 (-0.19, 0.58) | 0.32 |
| North American Native | 0.32 (-0.83, 1.47) | 0.58 | -0.30 (-1.29, 0.70) | 0.56 | 0.62 (0.04, 1.19) | 0.03 |
| Other/unknown | 0.81 (-0.50, 2.12) | 0.23 | -0.43 (-1.46, 0.60) | 0.41 | 1.24 (0.43, 2.05) | <0.001 |
| Cancer type |  |  |  |  |  |  |
| Breast | Ref |  | Ref |  | Ref |  |
| Colorectal | -0.09 (-0.41, 0.22) | 0.56 | -0.09 (-0.33, 0.16) | 0.49 | -0.01 (-0.20, 0.19) | 0.95 |
| Leukemia | -0.32 (-0.88, 0.25) | 0.27 | -0.58 (-0.96, -0.19) | 0.003 | 0.26 (-0.16, 0.67) | 0.23 |
| Lung | 0.20 (-0.14, 0.53) | 0.25 | -0.05 (-0.30, 0.21) | 0.72 | 0.25 (0.03, 0.46) | 0.03 |
| Lymphoma | 0.18 (-0.27, 0.64) | 0.43 | 0.06 -0.30, 0.41) | 0.75 | 0.13 (-0.16, 0.41) | 0.39 |
| Prostate | -0.05 (-0.39, 0.30) | 0.79 | -0.11 (-0.38, 0.16) | 0.44 | 0.06 (-0.16, 0.28) | 0.58 |
| Cancer stage |  |  |  |  |  |  |
| Localized | Ref |  | Ref |  | Ref |  |
| Regional | 0.46 (0.20, 0.72) | <0.001 | 0.50 (0.29, 0.71) | <0.001 | -0.04 (-0.19, 0.10) | 0.56 |
| Distant | 0.47 (0.20, 0.75) | 0.001 | 0.54 (0.32, 0.76) | <0.001 | -0.06 (-0.23, 0.11) | 0.47 |
| Unknown | -0.07 (-0.41, 0.26) | 0.67 | -0.11 (-0.32, 0.09) | 0.28 | 0.04 (-0.22, 0.31) | 0.75 |
| Previous MA coverage | -0.08 (-0.12, 0.04) | <0.001 | -0.08 (-0.12, -0.05) | <0.001 | 0.00 (-0.02, 0.02) | 0.84 |
| Diagnosis year |  |  |  |  |  |  |
| 2016 | 0.06 (-0.17, 0.29) | 0.58 | 0.03 (-0.16, 0.22) | 0.76 | 0.03 (-0.10, 0.17) | 0.61 |
| 2017 | -0.10 (-0.32, 0.12) | 0.36 | -0.13 (-0.30, 0.05) | 0.15 | 0.02 (-0.11, 0.16) | 0.72 |
| Constant | 3.10 (1.85, 4.34) | <0.001 | 2.89 (1.81, 3.98) | <0.001 | 0.20 (-0.42, 0.82) | 0.52 |
| Observations | 48,266 |  | 48,266 |  | 48,266 |  |
| R^2^ | 0.0562 |  | 0.0389 |  | 0.0769 |  |

**Source:** Author’s analysis of SEER-Medicare data, 2016-2019.

**Notes:** Beneficiary-level analysis. Outcomes measured one-month post cancer diagnosis. Results from linear probability models with robust standard errors and adjusted for age, race/ethnicity, sex, original reason for Medicare entitlement, cancer stage, cancer type, year and county fixed effects, and total months of previous MA enrollment the year before the beneficiary’s diagnosis. Old age and survivor’s insurance (OASI), Disability insurance benefit (DIB), End-stage renal disease (ESRD).

**Table 4.** Changes in Medicare enrollment two months after cancer diagnosis due to the extended Medicare Advantage Open Enrollment Period**,** 2016-2019

|  | Any Switch | | Disenrollment from  MA to TM | | Switch old MA plan to  new MA plan | |
| --- | --- | --- | --- | --- | --- | --- |
|  | Coeff (95% CI) | p-value | Coeff (95% CI) | p-value | Coeff (95% CI) | p-value |
| Diagnosis Month | Ref |  | Ref |  | Ref |  |
| April |  |  |  |  |  |  |
| January | 0.34 (-0.25, 0.93) | 0.26 | 0.35 (-0.22, 0.91) | 0.23 | -0.01 (-0.19, 0.17) | 0.94 |
| Post (2019) |  |  |  |  |  |  |
| No | Ref |  | Ref |  | Ref |  |
| Yes | -0.70 (-1.5, 0.13) | 0.10 | -0.70 (-1.48, 0.08) | 0.08 | 0.003 (-0.28,0.29) | 0.98 |
| April*post | Ref |  | Ref |  | Ref |  |
| January*post | 1.27 (0.20, 2.35) | 0.02 | 0.60 (-0.40, 1.60) | 0.24 | 0.67 (0.25, 1.09) | 0.002 |
| Age | 0.21 (0.16, 0.25) | <0.001 | 0.21 (0.17, 0.25) | <0.001 | -0.01 (-0.02,0.00) | 0.20 |
| Sex |  |  |  |  |  |  |
| Female | Ref |  | Ref |  | Ref |  |
| Male | 1.48 (0.53, 2.43) | 0.002 | 1.51 (0.59, 2.44) | 0.001 | -0.03 (-0.29,0.22) | 0.79 |
| Reason for Medicare Entitlement |  |  |  |  |  |  |
| OASI | Ref |  | Ref |  | Ref |  |
| DIB, ESRD, Both DIB& ESRD | 1.39 (0.57, 2.21) | 0.001 | 1.45 (0.68, 2.23) | <0.001 | -0.06 (-0.35, 0.22) | 0.67 |
| Race/ethnicity |  |  |  |  |  |  |
| Black | Ref |  | Ref |  | Ref |  |
| White | -1.31 (-3.34, 0.72) | 0.21 | -1.6 (-3.5, 0.41) | 0.12 | 0.26 (-0.21, 0.72) | 0.28 |
| Asian | -1.31 (-3.46, 0.83) | 0.23 | -2.1 (-4.1, 0.01) | 0.05 | 0.74 (0.17, 1.31) | 0.01 |
| Hispanic | -1.85 (-4.23, 0.53) | 0.13 | -2.1 (-4.4, 0.18) | 0.07 | 0.28 (-0.31, 0.86) | 0.35 |
| Native American | -0.42 (-2.91, 2.07) | 0.74 | -0.9 (-3.3, 1.5) | 0.48 | 0.44 (-0.22, 1.10) | 0.19 |
| Other/unknown | 0.50 (-2.23, 3.29) | 0.71 | -0.90 (-3.4, 1.6) | 0.48 | 1.43 (0.28, 2.58) | 0.01 |
| Cancer type |  |  |  |  |  |  |
| Breast | Ref |  | Ref |  | Ref |  |
| Colorectal | 0.98 (0.09, 1.9) | 0.03 | 0.93 (0.10, 1.8) | 0.03 | 0.04 (-0.27, 0.35) | 0.79 |
| Leukemia | -9.39 (-11.0, -7.8) | <0.001 | -9.8 (-11.2, -8.3) | <0.001 | 0.36 (-0.31, 1.04) | 0.29 |
| Lung | 5.94 (5.01, 6.9) | <0.001 | 5.8 (4.9, 6.6) | <0.001 | 0.19 (-0.14, 0.53) | 0.26 |
| Lymphoma | -0.14 (-1.52, 1.2) | 0.84 | -0.3 (-1.6, 1.0) | 0.64 | 0.17 (-0.29, 0.62) | 0.47 |
| Prostate | -2.23 (-3.28, -1.2) | <0.001 | -2.2 (-3.2, -1.2) | <0.001 | -0.06 (-0.40, 0.28) | 0.73 |
| Cancer Stage |  |  |  |  |  |  |
| Localized | Ref |  | Ref |  | Ref |  |
| Regional | 1.02 (0.44, 1.6) | 0.001 | 1.18 (0.64, 1.71) | <0.001 | -0.16 (-0.38, 0.07) | 0.17 |
| Distant | 10.21 (9.27, 11.1) | <0.001 | 10.34 (9.44, 11.24) | <0.001 | -0.14 (-0.41, 0.14) | 0.34 |
| Unknown | 2.97 (1.91, 4.0) | <0.001 | 2.89 (1.91, 3.87) | <0.001 | 0.08 (-0.33, 0.48) | 0.70 |
| Previous MA coverage | -0.15 (-0.23, -0.1) | 0.00 | -0.15 (-0.23, -0.07) | <0.001 | 0.00 (-0.03, 0.03) | 0.86 |
| Diagnosis Year |  |  |  |  |  |  |
| year_2016 | 0.38 (-0.35, 1.1) | 0.31 | 0.44 (-0.27, 1.14) | 0.224 | -0.06 (-0.28, 0.17) | 0.62 |
| year_2017 | -0.15 (-0.84, 0.5) | 0.67 | -0.06 (-0.72, 0.60) | 0.850 | -0.09 (-0.30, 0.13) | 0.43 |
| Constant | -11.3 (-14.8, -7.7) | <0.001 | -12.0 (-15.5, -8.61) | <0.001 | 0.77 (-0.17, 1.71) | 0.11 |
| Observations | 31,785 |  | 31,785 |  | 31,785 |  |
| R^2^ | 0.1169 |  | 0.1191 |  | 0.0920 |  |

**Source:** Author’s analysis of SEER-Medicare data, 2016-2019.

**Notes:** Beneficiary-level analysis. Outcomes measured two-months post cancer diagnosis. Results from linear probability models with robust standard errors and adjusted for age, race/ethnicity, sex, original reason for Medicare entitlement, cancer stage, cancer type, year and county fixed effects, and total months of previous MA enrollment the year before the beneficiary’s diagnosis. Old age and survivor’s insurance (OASI), Disability insurance benefit (DIB), End-stage renal disease (ESRD).
